# Vitamin D_3_ oral rinsing reduces salivary SARS-CoV-2 antigen detectability: a clinical trial with public health implications

**DOI:** 10.3389/froh.2026.1943222

**Published:** 2026-09-01

**Authors:** Sarah M. Nizar Feteih, Emily L. G. Heaphy, Amjad Aljagthmi, Abdullah Abumdaini, Daniyah Bayumi, Ghadeer Albishi, Ashraf Dada

**Affiliations:** 1 King Faisal Specialist Hospital and Research Centre, Department of Dentistry, Periodontology, Jeddah, Saudi Arabia; 2 King Faisal Specialist Hospital and Research Centre, Department of Research Center, Jeddah, Saudi Arabia; 3 King Faisal Specialist Hospital and Research Centre, Department of Pharmacy, Jeddah, Saudi Arabia; 4 King Faisal Specialist Hospital and Research Centre, Department of Pathology and Laboratory Medicine, Virology Technology, Jeddah, Saudi Arabia; 5 Al-Faisal University, College of Medicine, Riyadh, Saudi Arabia

**Keywords:** pre-analytical interference, public health surveillance, rapid antigen test (RAT), saliva, SARS-CoV-2, vitamin D oral rinse

## Abstract

**Objectives:**

To determine whether a Vitamin D_3_ oral rinse alters the performance of saliva-based SARS-CoV-2 rapid antigen testing (RAT) and assess its potential implications for infectious disease screening and public health surveillance.

**Methods:**

In this prospective, paired-sample, single-blind clinical trial, 25 adults with laboratory-confirmed SARS-CoV-2 infection provided saliva immediately before and after a standardized one-minute Vitamin D₃ oral rinse (50 paired specimens). Samples were analyzed in parallel by saliva RAT and rapid RT-PCR under blinded conditions.

**Results:**

The oral rinse converted 68% (17/25) of paired specimens from RAT-positive to RAT-negative, reducing antigen positivity from 54.2% to 32.0% (*p* = 0.0016). In contrast, RT-PCR positivity remained largely preserved (96.0% vs. 88.0%), with no significant change in mean Ct values (28.1 ± 5.9 vs. 29.4 ± 5.1; *p* = 0.1785). Strong concordance between paired RT-PCR samples (*ρ* = 0.814, *p* < 0.0001) indicated persistent viral RNA despite reduced antigen detection.

**Conclusions:**

Vitamin D₃ oral rinsing was associated with reduced detection by saliva-based SARS-CoV-2 RAT while RT-PCR results remained largely preserved. These findings suggest a previously under-recognized pre-analytical variable capable of altering saliva-based antigen detection.

**Clinical Trial Registration:** https://clinicaltrials.gov/study/NCT07068282, Identifier, NCT07068282.

## Background

Saliva-based diagnostic testing has fundamentally transformed COVID-19 surveillance capacity in public health systems globally (1). Compared to nasopharyngeal sampling, saliva collection offers distinct advantages (2) for population-level screening: it is non-invasive, patient-acceptable, amenable to self-administration, and suitable for decentralized testing in community settings, dental offices, and emergency departments (3, 4). Thus, rapid antigen tests performed on saliva specimens have become central to COVID-19 surveillance (5) infrastructure in many countries, enabling mass screening and rapid case identification (6, 7). However, this transition to saliva-based diagnostics has created a critical but largely unrecognized vulnerability: inadequate standardization of the pre-collection phase (8). The pre-analytical phase, encompassing all procedures before specimen analysis, accounts for 46%–60% of diagnostic errors in clinical laboratories (9), yet remains poorly standardized in most saliva-based testing programs. This discrepancy is particularly concerning for SARS-CoV-2, where rapid antigen testing serves as the primary screening tool in surveillance programs. Systematic interference with test accuracy directly compromises public health decision-making, from individual patient isolation recommendations to population-level infection control strategies and outbreak detection.

In clinical practice, patients present for testing after diverse oral behaviors: use of mouthwashes, dentifrices, foods, and beverages. Despite the increasing adoption of saliva-based diagnostics, the potential influence of commonly used oral products on saliva antigen detection remains poorly characterized. This represents an important gap in the standardization of pre-collection procedures and may contribute to variability in diagnostic performance (8).

A particularly relevant development is the recent market availability of vitamin D3 oral rinses marketed for dental remineralization (6) and immune enhancement (10). Unlike traditional antiseptic mouthwashes that function through direct virucidal mechanisms (11), vitamin D3-based products operate through immunomodulatory pathways (12, 13). Vitamin D₃ regulates the expression of antimicrobial peptides and contributes to innate immune defense within the oral cavity (14, 15). Its influence on salivary composition may alter the biochemical environment of saliva, providing a plausible explanation for the reduced antigen detectability observed in the present study (16). However, this proposed mechanism remains speculative and warrants further investigation.

Our previous laboratory study evaluating the effects of novel vitamin D₃ compounds on saliva samples from patients with COVID-19 provided the rationale for the present prospective clinical investigation (10). Building on these findings, we conducted the present prospective clinical trial to determine whether the previously demonstrated *in vitro* interference mechanisms translate into diagnostic interference in clinical specimens. Consequently, this prospective study was designed, specifically to: (1) quantify Vitamin D₃-induced diagnostic interference in patient saliva specimens, (2) explore the potential explanations underlying this interference, (3) assess the real-world prevalence of pre-collection standardization across diverse laboratory settings, and (4) consider the potential public health implications of pre-analytical interference.

## Methods

Study design, setting, and ethics: This prospective, single-center, interventional clinical trial was conducted at King Faisal Specialist Hospital & Research Centre in Jeddah, Saudi Arabia, a 500-bed tertiary referral facility serving a patient population of approximately 2 million in Jeddah and the western region of Saudi Arabia. The study was approved by the Institutional Review Board (IRB #2022-32), registered with the Saudi Food and Drug Authority (SFDA #23041602), and prospectively registered at ClinicalTrials.gov (NCT07068282). All procedures adhered to the Declaration of Helsinki, and written informed consent was obtained from all participants prior to enrollment.

Sample Size Justification: This study was designed as an exploratory pilot clinical trial. The planned sample size was determined in consultation with a biostatistician and was considered appropriate for a pilot interventional study based on feasibility and the objective of generating preliminary data. Therefore, no formal *a priori* sample size calculation was performed.

Eligible participants were symptomatic outpatients with laboratory-confirmed SARS-CoV-2 infection diagnosed during their clinical visit. Whenever feasible, study enrollment and saliva collection were performed on the same day as diagnostic confirmation; the 48 h interval represented the maximum allowable time between diagnosis and study enrollment to accommodate logistical considerations.

**Figure 1:**
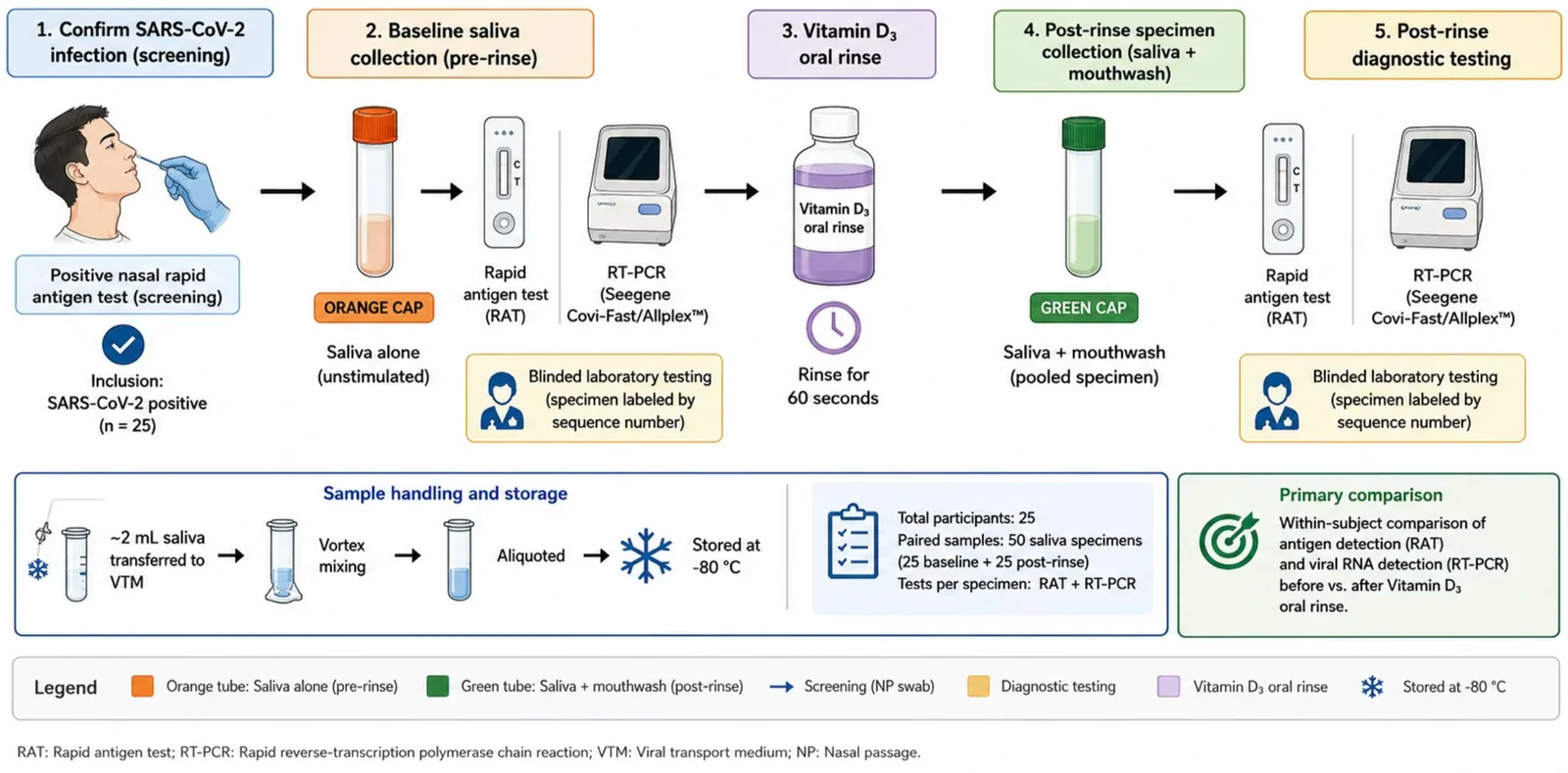
Study design and prospective paired-sample clinical trial workflow.

Study population and specimen collection: Inclusion criteria were: (1) age ≥18 years; (2) laboratory confirmation of active SARS-CoV-2 infection by nasal rapid antigen testing within 48 h; (3) written informed consent. Exclusion criteria were: (1) immunocompromised status; (2) pregnancy; (3) age <18 years; and (4) concurrent prescription vitamin D supplementation ≥10,000 IU daily. Twenty-five COVID-19 patients completed the protocol. Two unstimulated whole saliva specimens were collected from each participant: Specimen 1 (pre-rinse baseline) prior to oral intervention into a sterile tube with 2 mL viral transport medium; Specimen 2 (post-rinse) after immediate rinsing mouth with 1–2 mL vitamin D3 formulation for 60 s, then collecting into an identical tube. All specimens were coded with participant identifiers and processed identically. Importantly, laboratory personnel performing testing were blinded to specimen sequence. Figure 1 shows the study design and specimen collection workflow.

Twenty-five adults with laboratory-confirmed SARS-CoV-2 infection each provided two paired saliva specimens. An unstimulated baseline saliva specimen was collected before oral rinsing and later analyzed by rapid antigen testing (RAT) and rapid reverse-transcription polymerase chain reaction (RT-PCR). Approximately 15–30 min later, participants received the investigational Vitamin D₃ oral rinse, swished for 60 s, and then passively drooled the pooled saliva–mouthwash specimen into a second sterile collection tube.

Vitamin D₃ oral rinse: The investigational oral rinse was compounded by the Department of Pharmacy at King Faisal Specialist Hospital & Research Centre (Jeddah, Saudi Arabia) under standardized aseptic conditions. The formulation contained cholecalciferol (vitamin D₃, as the main active ingredient) as the active ingredient. Because the formulation is subject to institutional intellectual property protection, the excipient composition, vehicle, and detailed preparation procedure are not disclosed. To ensure product integrity, minimize the risk of cross-contamination, and maintain dosing consistency, each participant received an individually dispensed light-protective vial prepared exclusively for single-patient use. The formulation was stored at 4°C under light-protected conditions. Based on institutional pharmacy recommendations, each preparation batch was assigned a shelf life of 4–6 weeks, after which a freshly compounded batch was prepared for subsequent participant recruitment.

Blinding: Because of the nature of the intervention, participants were aware that they received the investigational vitamin D_3_ oral rinse and were therefore not blinded. The principal investigator maintained the specimen identification key and was the only individual aware of specimen identity and collection sequence throughout sample collection. Laboratory personnel performing and interpreting the rapid antigen test and RT-PCR assays remained blinded to specimen identity, collection sequence, and participant information throughout testing and interpretation. Specimen allocation was disclosed only after all laboratory analyses had been completed.

Specimen collection: Baseline unstimulated whole saliva was first collected into a sterile tube by passive drooling. After an interval of approximately 15–30 min, participants received 1–2 mL of the investigational vitamin D_3_ oral formulation and were instructed to swish the solution throughout the oral cavity for 60 s. At the end of the rinsing period, participants gently drooled the oral rinse–saliva mixture into a second sterile collection tube. Both specimens were handled and processed identically.

Testing methodologies and quality assurance: Rapid antigen detection was performed blindly by color coding the samples by a lab technician using Singuway Colloidal Gold Cassette and SARS-CoV-2 Rapid Antigen Test 2.0 (Roche) per manufacturer protocols, with each specimen undergoing 2–3 independent test iterations. All saliva specimens underwent real-time reverse-transcription PCR using the Xpert® Xpress CoV-2/Flu/RSV Plus assay (Cepheid, Sunnyvale, CA, USA). Cycle threshold (Ct) values were recorded for all positive results. Paired Student's t-tests compared mean Ct values between pre-rinse and post-rinse specimens (17). McNemar's test assessed changes in binary rapid antigen test outcomes (17, 18). Pearson correlation evaluated paired RT-PCR relationship.

To estimate population-level impact, we applied observed false-negative rates to WHO COVID-19 testing volume using published surveillance data (19). Public health implications were assessed through review of previous publications on COVID-19 diagnostic failures and infection control (13–15).

**Figure 2:**
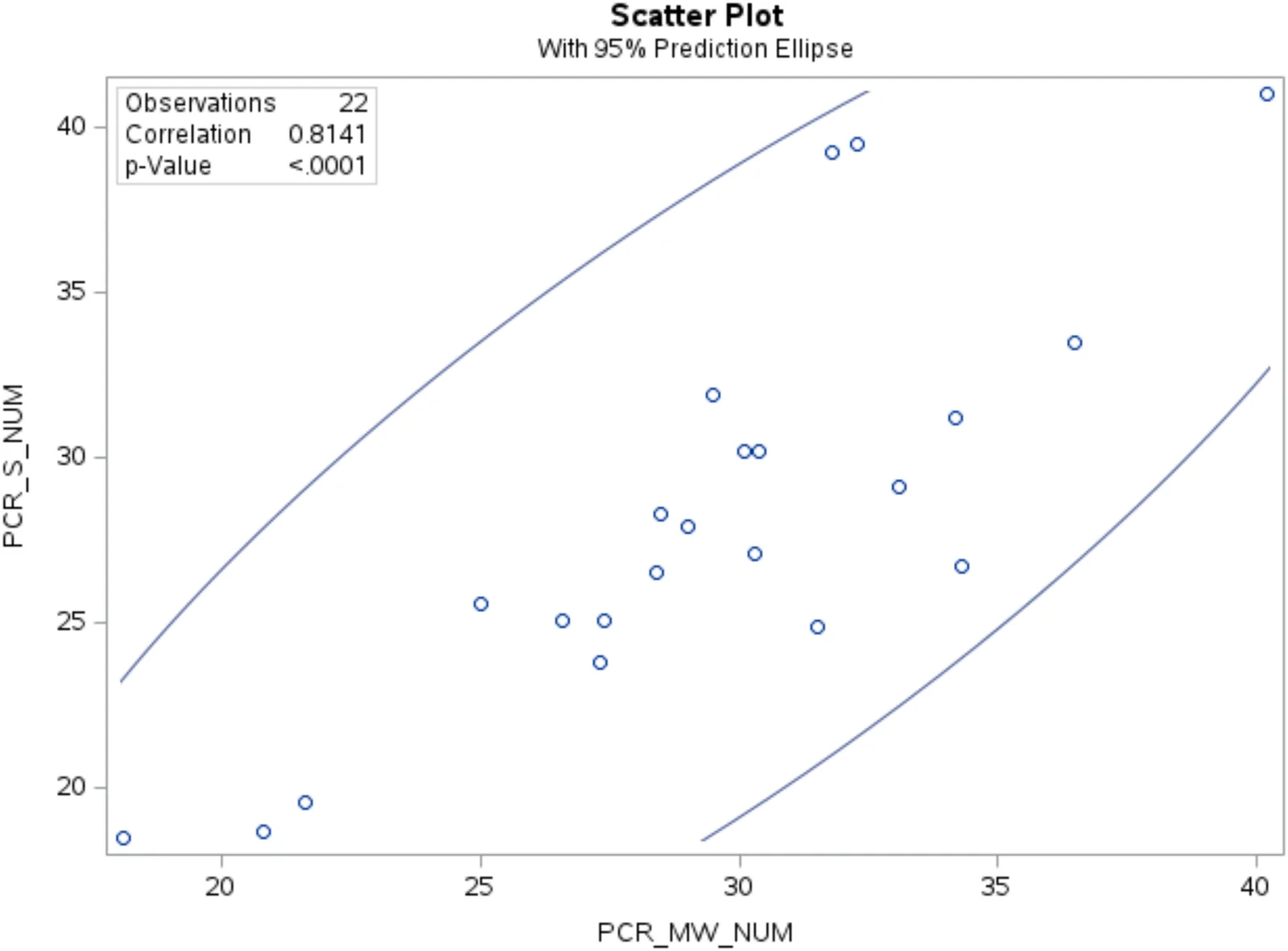
Correlation between pre-rinse and post-rinse RT-PCR cycle threshold (Ct) values (*n* = 22).

Statistical analysis: Continuous variables were summarized as mean ± standard deviation (SD), whereas categorical variables were expressed as frequencies and percentages. The distribution of continuous variables was assessed using the Shapiro–Wilk test for normality. Associations between clinical variables were evaluated using paired Student's *t*-tests to compare RT-PCR Ct values before and after mouthwash collections, independent-samples *t*-tests to compare Ct values according to RAT status, McNemar's test for paired categorical outcomes, and Pearson's correlation coefficient to assess the linear association between paired RT-PCR Ct values. Samples yielding RT-PCR error results or without measurable Ct values were excluded from Ct-based analyses. Statistical analyses were performed using SAS Studio, and statistical significance was defined as *α* = 0.05. The archived statistical documentation available to the authors specifies the statistical methods, software, and significance threshold but does not explicitly indicate whether the analyses were one-sided or two-sided. We therefore elected not to state this in the revised manuscript rather than include information that we could not verify. Pearson correlation analysis revealed a strong positive linear association between RT-PCR Ct values from pre-rinse and post-rinse specimens (*ρ* = 0.814, *p* < 0.0001; Table 1). RT-PCR Ct values increased linearly across both specimen types, confirming the internal consistency of viral RNA measurements in paired specimens and further supporting the conclusion that the observed RAT interference reflects a pre-analytical effect rather than a change in viral abundance. This linear relationship is illustrated with a 95% prediction ellipse in Figure 2.

**Table 1:** Statistical associations between clinical variables and diagnostic test results (*n* = 25).

Comparison	Statistic	df	*p*-value
Difference in RT-PCR Ct values: saliva only vs. saliva with oral rinse (*n* = 22)	1.39	21	0.1785
RT-PCR Ct values by RAT result in saliva only specimens (positive vs. negative, *n* = 23)	2.07	21	0.0508
RT-PCR Ct values by RAT result in saliva with oral rinse specimens (positive vs. negative, *n* = 22)	2.83	20	0.0104
Concordance between RT-PCR and RAT results: saliva only specimens	10.00	1	0.0016
Concordance between RT-PCR and RAT results: saliva with oral rinse specimens	14.00	1	0.0002
Pearson correlation: RT-PCR Ct values, saliva only vs. saliva with oral rinse	*ρ* = 0.814	—	<0.0001

Statistical tests applied: Student's paired t-test (RT-PCR Ct value comparison); independent samples t-test (RT-PCR Ct values by RAT result); McNemar's test (RT-PCR vs. RAT concordance); Pearson's correlation. df, degrees of freedom; RT-PCR, rapid reverse-transcription polymerase chain reaction; RAT, rapid antigen test; ρ, Pearson correlation coefficient.

## Results

### Study population and specimen overview

A total of 25 adults with confirmed SARS-CoV-2 infection were enrolled. Two saliva specimens were collected per participant, one immediately prior to (pre-rinse, orange tube) and one following (immediate saliva-oral rinse, green tube) Vitamin D₃ oral rinse administration, yielding 50 specimens in total. Baseline diagnostic test results for all participants are summarized in Table 2. Full individual rapid RT-PCR cycle threshold (Ct) values for all 25 paired specimens are presented in Table 3.

**Table 2:** Diagnostic test results for all 25 participants (50 paired saliva specimens).

Variable	*n* (%)	Mean ± SD
RT-PCR: Saliva Only — Pre-rinse (Orange Tube)		
Mean Ct value	—	28.1 ± 5.9
Positive	24 (96.0)	—
Negative	1 (4.0)	—
Error	0 (0.0)	—
RT-PCR: Saliva with Vitamin D₃ Oral Rinse — Post-rinse (Green Tube)		
Mean Ct value	—	29.4 ± 5.1
Positive	22 (88.0)	—
Negative	1 (4.0)	—
Error	2 (8.0)	—
RAT: Saliva Only — Pre-rinse (Orange Tube)		
Positive	13 (54.2)	—
Negative	11 (45.8)	—
RAT: Saliva with Vitamin D_3_ Oral Rinse — Post-rinse (Green Tube)		
Positive	8 (32.0)	—
Negative	17 (68.0)	—

Two specimens were collected per participant, yielding 50 specimens in total. Orange tubes: saliva only, collected prior to oral rinse administration (pre-rinse, control). Green tubes: saliva collected following Vitamin D_3_ oral rinse administration (post-rinse, test). Ct, cycle threshold; RT-PCR, rapid reverse-transcription polymerase chain reaction; RAT, rapid antigen test; SD, standard deviation; -, not applicable.

**Table 3:** Individual RT-PCR cycle threshold (Ct) values for all 25 paired saliva specimens.

Specimen No.	Saliva Only — Pre-rinse (Orange Tube)		Saliva with Oral Rinse — Post-rinse (Green Tube)	
	RT-PCR Result	Ct Value	RT-PCR Result	Ct Value
1	Positive	34.2	Positive	31.2
2	Positive	28.4	Positive	26.5
3	Negative	N/A	Negative	N/A
4	Positive	30.3	Positive	27.1
5	Positive	28.5	Positive	28.3
6	Positive	29.0	Positive	27.9
7	Positive	26.6	Positive	25.1
8	Positive	21.6	Positive	19.6
9	Positive	29.5	Positive	31.9
10	Positive	27.3	Positive	23.8
11	Positive	30.4	Positive	30.2
12	Positive	31.5	Positive	24.9
13	Error	—	Positive	25.4
14	Positive	18.1	Positive	18.5
15	Positive	30.1	Positive	30.2
16	Positive	34.3	Positive	26.7
17	Positive	27.4	Positive	25.1
18	Positive	20.8	Positive	18.7
19	Positive	32.3	Positive	39.5
20	Positive	33.1	Positive	29.1
21	Positive	36.5	Positive	33.5
22	Positive	40.2	Positive	41.0
23	Positive	31.8	Positive	39.2
24	Error	—	Positive	24.7
25	Positive	25.0	Positive	25.6

Orange tubes: saliva collected prior to Vitamin D₃ oral rinse administration (pre-rinse, control specimen). Green tubes: saliva collected following oral rinse administration (post-rinse, test specimen). Error: specimen processing error; excluded from statistical analysis. N/A: not applicable (specimen tested negative; no Ct value generated). RT-PCR, rapid reverse-transcription polymerase chain reaction.

### Reverse-transcription PCR findings

Pre-rinse rapid RT-PCR positivity was 96.0% (24/25 specimens). Following oral rinse passive drooling collection, RT-PCR positivity was 88.0% (22/25), with two post-rinse specimens returning error results and one returning a negative result. Mean Ct values were 28.1 ± 5.9 for pre-rinse specimens and 29.4 ± 5.1 for post-rinse specimens. This difference was not statistically significant (*t* = 1.39, df = 21, *p* = 0.1785; Table 1), indicating that Vitamin D₃ oral rinsing did not meaningfully reduce viral RNA detectability.

Among pre-rinse specimens, mean RT-PCR Ct values did not differ significantly between RAT-positive and RAT-negative results (25.8 vs. 30.7; *t* = 2.07, df = 21, *p* = 0.0508). In contrast, among post-rinse specimens, mean Ct values differed significantly between RAT-positive and RAT-negative results (25.9 vs. 31.4; *t* = 2.83, df = 20, *p* = 0.0104; Table 1), indicating that post-rinse RAT negativity was associated with relatively higher Ct values, consistent with antigen concentration falling below the assay detection threshold following oral rinsing.

### Rapid antigen test findings

**Figure 3:**
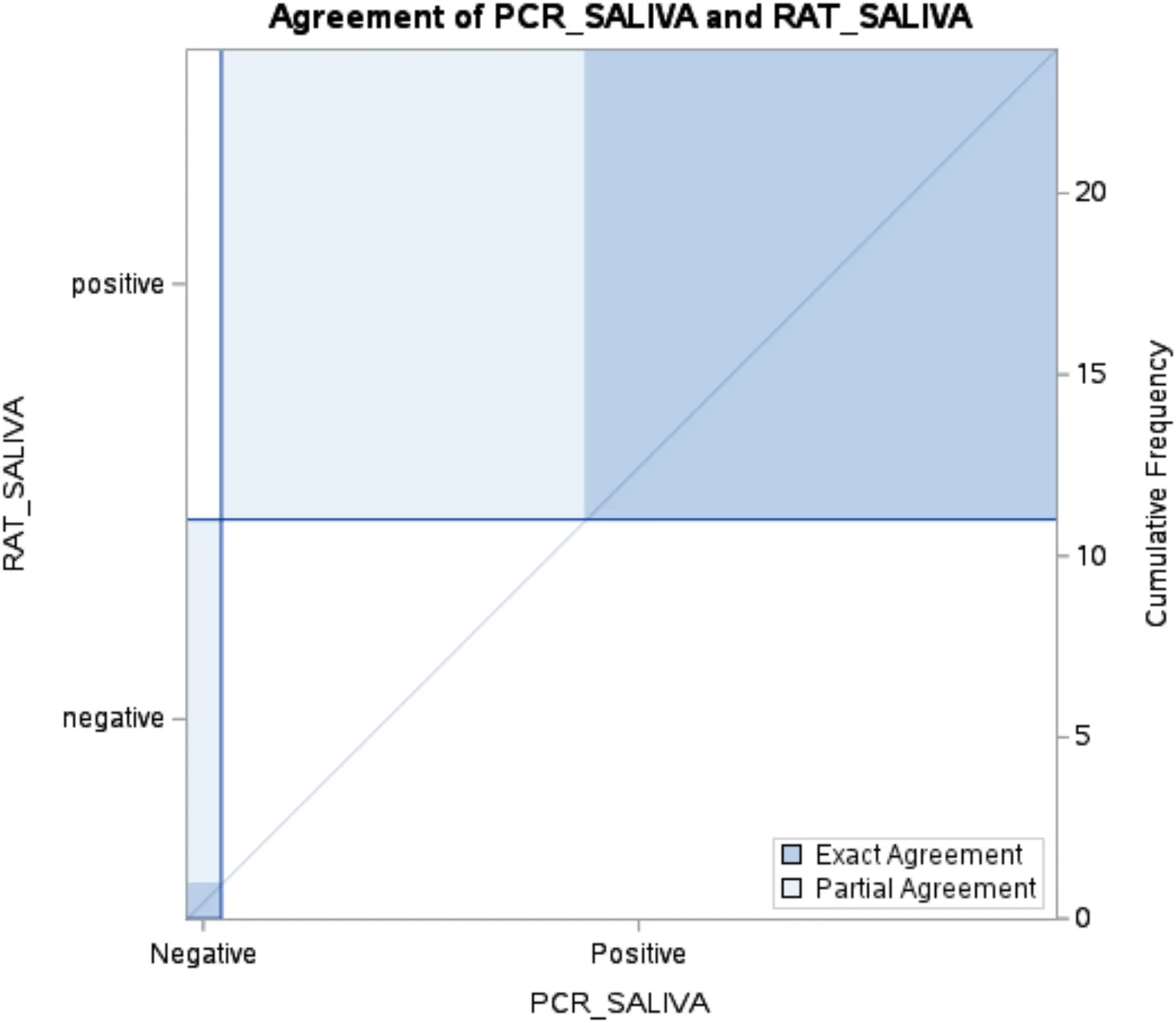
Agreement between rapid antigen testing (RAT) and rapid reverse-transcription polymerase chain reaction (RT-PCR) results in baseline saliva specimens (*n* = 25).

**Figure 4:**
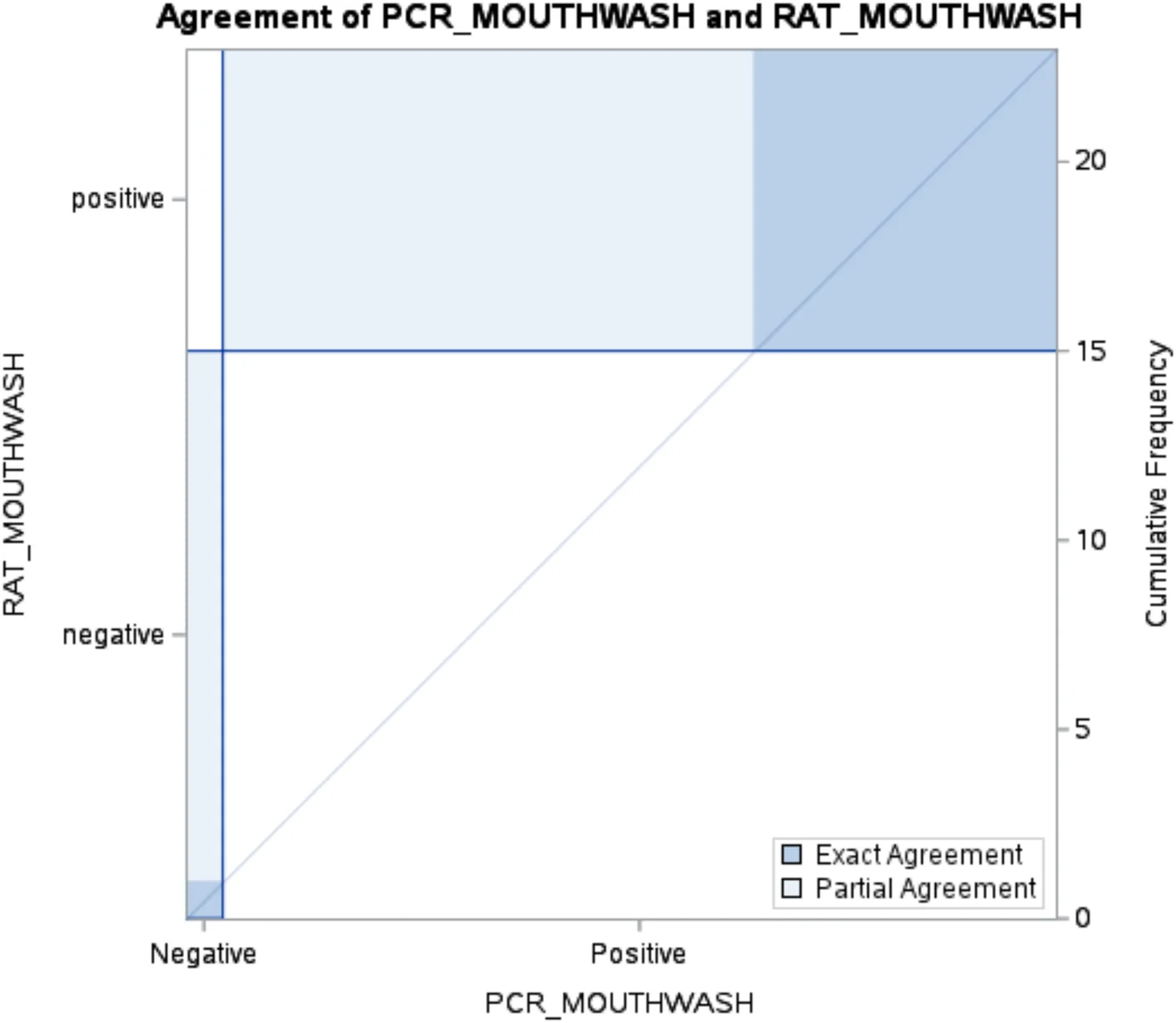
Agreement between rapid antigen testing (RAT) and rapid reverse-transcription polymerase chain reaction (RT-PCR) results following vitamin D_3_ oral rinsing (*n* = 25).

Pre-rinse RAT positivity was 54.2% (13 of 24 valid specimens). Following Vitamin D₃ oral rinse administration, RAT positivity declined to 32.0% (8/25), with 68% of post-rinse specimens (17/25) converting to a negative result. Comparison of RT-PCR and RAT results using McNemar's test demonstrated a statistically significant discordance between the two diagnostic modalities for both pre-rinse specimens (*p* = 0.0016) and post-rinse specimens (*p* = 0.0002; Table 1), confirming that the choice of diagnostic method significantly influenced test outcome. Graphical comparisons of RT-PCR and RAT results for pre-rinse and post-rinse specimens are presented in Figures 3, 4, respectively. The pattern of post-rinse RAT conversion to negative occurred in the presence of persistently detectable viral RNA. These findings suggest selective interference with antigen detectability while viral RNA detection remained largely preserved.

Agreement plot comparing RAT and RT-PCR results obtained from saliva specimens collected before oral rinsing. The plot demonstrates the concordance between the two diagnostic methods in baseline saliva samples, showing higher detection by RT-PCR than by RAT.

Agreement plot comparing RAT and RT-PCR results obtained from saliva specimens collected immediately after Vitamin D₃ oral rinsing. Compared with baseline specimens, RT-PCR positivity remained largely preserved, whereas RAT positivity decreased, suggesting selective interference with antigen detectability rather than viral RNA detection.

Scatter plot illustrating the relationship between baseline (pre-rinse) RT-PCR Ct values (*x*-axis) and post-rinse RT-PCR Ct values (*y*-axis) obtained from paired saliva specimens with measurable Ct values. Each point represents one participant. Pearson correlation analysis revealed a strong positive association (*r* = 0.814, *p* < 0.0001), indicating that RT-PCR Ct values remained highly consistent before and after Vitamin D₃ oral rinsing despite the observed reduction in rapid antigen test positivity.

### Adverse events

No side effects or adverse events were reported by any participant at any time during the study.

## Discussion

This study identifies a previously under-recognized source of pre-analytical variability in saliva-based SARS-CoV-2 antigen testing. The significant reduction of 68% in rapid antigen test positivity (*p* = 0.0016) following vitamin D3 oral rinsing suggests a systematic pre-analytical effect on salivary antigen detectability under the conditions evaluated. This finding is mechanistically distinct from traditional antiseptic mouthwashes, which function through direct virucidal mechanisms (20–22). RT-PCR findings did not demonstrate a significant change in viral RNA detection following rinsing. Although a trend toward lower RT-PCR Ct values was observed among pre-rinse RAT-positive specimens, this difference did not reach conventional statistical significance (*p* = 0.0508) and should therefore be interpreted with caution. These findings suggest that the observed reduction in antigen positivity was not accompanied by a corresponding measurable reduction in viral RNA under the conditions studied.

Vitamin D₃ exerts immunomodulatory effects on mucosal epithelial environments (10), including modulation of antimicrobial peptides and salivary immune proteins (23), which may plausibly influence antigen detectability without affecting viral RNA stability (24–26). The clinical significance of these findings is amplified by evolving market trends, including a booming industry of incorporation of vitamin D into consumer oral care formulations and the widespread marketing of dental products with immune-related claims (27).

Published evidence demonstrates substantial heterogeneity in pre-analytical workflows for saliva-based SARS-CoV-2 testing, with no universally standardized protocol for specimen collection, handling, or stabilization across clinical and surveillance settings (28, 29). Unlike nasopharyngeal swab protocols, which are widely standardized, saliva pre-collection guidance varies substantially or is absent in many real-world settings (30–32). This absence carries measurable consequences: a hospital-based study demonstrated that patients frequently arrive for testing and staff are unaware of the diagnostic impact of routine pre-collection mouth washing on saliva-based SARS-CoV-2 detection (33). The outcome of our clinical findings creates a real-world challenging scenario where diagnostic interference occurs undetected. These findings emphasize the importance of standardized saliva specimen collection and pre-collection protocols (34) to minimize variability and improve the consistency of saliva-based diagnostic testing. These clinical observations extend our research trajectory that began with laboratory identification of the interference mechanism (35) and now reaches prospective validation in a controlled clinical setting. These findings, which align with and extend previous investigations of diagnostic interference in saliva-based testing (4, 36), reinforce the importance of standardized pre-collection procedures for ensuring the quality of saliva-based testing programs.

This study employed a paired within-subject design in which each participant served as their own control. Accordingly, the observed reduction in RAT positivity after the vitamin D₃ mouthwash should be interpreted as a change in antigen detectability within the same individual rather than evidence of true diagnostic failure. Because all participants were laboratory-confirmed SARS-CoV-2 infection at baseline, the study was not designed to estimate diagnostic sensitivity or specificity in the conventional sense. Instead, the findings suggest the potential influence of a pre-analytical oral intervention on saliva-based antigen detection while demonstrating that RT-PCR detection remained largely preserved.

The findings of this pilot study should be interpreted within the context of saliva-based SARS-CoV-2 testing, where pre-analytical factors may influence diagnostic performance. As saliva continues to be evaluated as a convenient and non-invasive specimen for infectious disease testing, standardization of specimen collection and pre-collection conditions is increasingly important. Although this study was not designed to evaluate population-level diagnostic performance, the observed reduction in rapid antigen detectability suggests that recent oral interventions, including mouth rinsing, may represent a source of pre-analytical variability that should be considered when developing saliva collection protocols to optimize the consistency and interpretation of saliva-based antigen testing.

The present findings suggest that recent oral interventions, including mouth rinsing, should be considered when developing standardized saliva collection protocols to optimize the consistency and interpretation of saliva-based antigen testing. If supported by larger multicenter studies, these findings may help inform future institutional protocols and clinical practice guidelines governing saliva-based diagnostic testing, particularly in settings where non-invasive specimen collection is preferred.

Future multicenter studies involving larger cohorts and appropriate control groups are warranted to evaluate different oral rinse formulations, concentrations, collection protocols, sampling intervals, and diagnostic testing conditions. Such studies will further clarify the influence of pre-analytical oral interventions on saliva-based diagnostic performance and help inform evidence-based pre-collection recommendations.

### Study limitations

The present findings also have broader implications for the continued integration of saliva-based diagnostics into clinical practice. Saliva offers several advantages over conventional sampling methods, including its non-invasive nature, ease of collection, improved patient acceptability, and suitability for repeated testing. These characteristics make saliva particularly attractive in clinical settings where conventional specimen collection may be less desirable or more burdensome for patients. As healthcare systems increasingly adopt patient-centered diagnostic approaches, ensuring standardized pre-collection conditions becomes an important component of test reliability. The present findings suggest that recent oral interventions, including mouth rinsing, should be considered when developing standardized saliva collection protocols. If confirmed in larger multicenter studies, these findings may help inform future institutional policies and clinical practice guidelines governing saliva-based diagnostic testing.

Several limitations should be acknowledged. First, this was a pilot single-center study with a relatively small sample size, which may limit the generalizability of the findings. Second, only one vitamin D₃ oral rinse formulation, one rinse volume, one swishing duration (60 s), and one post-rinse collection protocol were evaluated. Consequently, the findings should not be extrapolated to other formulations, concentrations, rinse durations, or collection intervals. Third, the study evaluated the immediate effect of the intervention in a paired within-subject design and was not intended to investigate the duration of any observed effect or the underlying biological mechanisms. Finally, because all participants had confirmed COVID-19 infection, the study was not designed to determine conventional diagnostic accuracy measures in a broader screening population. Future studies involving larger, multicenter cohorts should evaluate different vitamin D₃ concentrations, rinse volumes, swishing durations, and sampling intervals to determine whether the observed changes in antigen detectability persist over time or vary according to collection conditions.

An additional limitation is the absence of a comparator oral rinse group, which precluded direct comparison of the observed effects with those of saline, placebo, or commercially available mouthwashes. Accordingly, definitive attribution of the observed reduction in antigen detectability to the vitamin D₃ formulation itself is not possible, as mechanical effects of oral rinsing (e.g., dilution or removal of salivary antigens) cannot be excluded. Future studies incorporating comparator rinses would help distinguish formulation-specific effects from broader pre-analytical influences on saliva-based diagnostics.

An incidental delayed color change was observed in some post-rinse saliva specimens during sample processing. Although this observation was not prospectively documented or systematically evaluated and therefore cannot be interpreted further, reporting it may encourage future mechanistic investigations into potential physicochemical interactions between vitamin D formulations, saliva constituents, and antigen detection assays.

The prospective paired-sample design, blinded testing protocol, and standardized specimen collection procedures nonetheless strengthen the robustness of the findings.

These findings warrant confirmation in larger multicenter studies involving diverse patient populations and comparator oral rinse formulations.

## Conclusions

This study identifies a previously under-recognized source of pre-analytical variability in saliva-based SARS-CoV-2 rapid antigen testing associated with prior use of a vitamin D₃-fortified oral rinse. Antigen detection was reduced following oral rinse exposure, while RT-PCR results remained largely stable, suggesting an effect on antigen detectability rather than viral RNA presence.

These findings highlight the importance of pre-collection conditions as a determinant of diagnostic performance in saliva-based testing. However, current variability in saliva collection and testing workflows across clinical settings limits the ability to generalize findings across all diagnostic environments.

As saliva-based diagnostics continue to expand due to their acceptability and feasibility in populations where nasopharyngeal sampling may be challenging, these results support the need for further systematic evaluation of pre-analytical variables across diverse and real-world testing settings. Such evidence will be essential for informing future harmonization efforts and for guiding the development of standardized, context-sensitive protocols that can support both clinical implementation and public health surveillance.

## Data Availability

The data presented in the study are included in the article/supplementary material, further inquiries can be directed to the corresponding author/s.
